# Are we doing enough? Improved breastfeeding practices at 14 weeks but challenges of non-initiation and early cessation of breastfeeding remain: findings of two consecutive cross-sectional surveys in KwaZulu-Natal, South Africa

**DOI:** 10.1186/s12889-020-08567-y

**Published:** 2020-04-03

**Authors:** C. Horwood, L. Haskins, I. Engebretsen, C. Connolly, A. Coutsoudis, L. Spies

**Affiliations:** 1 Centre for Rural Health, University of KwaZulu-Natal, Durban, South Africa; 2 Centre for International Health, Department of Global Public Health and Primary Care, University of Bergen, Bergen, Norway; 3 Department of Paediatrics & Child Health, School of Clinical Medicine, Nelson R Mandela School of Medicine, University of KwaZulu-Natal Durban, Durban, South Africa; 4 Department of Health, Pietermaritzburg, KwaZulu-Natal South Africa

**Keywords:** Exclusive breastfeeding, Breastfeeding, Child health, Infant feeding, Risk factors, HIV infection, Working women, South Africa

## Abstract

**Background:**

KwaZulu-Natal (KZN) Initiative for breastfeeding support (KIBS) was a multipronged intervention to support the initiation and sustaining of breastfeeding, implemented between 2014 and 2017. We present results of two surveys conducted before and after KIBS implementation to assess changes in infant feeding practices in KZN over this time period.

**Methods:**

Two cross-sectional surveys were conducted in primary health care clinics. Multistage stratified random sampling was used to select clinics and participants. Sample size was calculated to provide district estimates of 14-week exclusive breastfeeding (EBF) rates at baseline (KIBS1), and provincial estimates at endpoint (KIBS2). At KIBS1 the sample required was nine participating clinics in each of 11 districts (99 clinics) with 369 participants per district (*N* = 4059), and at KIBS2 was 30 clinics in KZN with 30 participants per clinic (*N* = 900). All caregivers aged ≥15 years attending the clinic with infants aged 13- < 16 weeks were eligible to participate. Data was collected using structured interviews on android devices. Multi-variable logistic regression was used to adjust odds ratios for differences between time points.

**Results:**

At KIBS1 (May2014- March2015), 4172 interviews were conducted with carers, of whom 3659 (87.6%) were mothers. At KIBS2 (January–August 2017), 929 interviews were conducted which included 788 (84.8%) mothers. Among all carers the proportion exclusively breastfeeding was 44.6 and 50.5% (*p* = 0.1) at KIBS1 and KIBS2 respectively, but greater improvements in EBF were shown among mothers (49.9 vs 59.1: *p* = 0.02). There were reductions in mixed breastfeeding among all infants (23.2% vs 16.3%; *p* = 0.016). Although there was no change in the proportion of carers who reported not breastfeeding (31.9% vs 32.8%; *p* = 0.2), the duration of breastfeeding among mothers who had stopped breastfeeding was longer at KIBS2 compared to KIBS1 (*p* = 0.0015). Mothers who had returned to work or school were less likely to be breastfeeding (adjusted odds ratio [AOR] 3.76; 95% CI 3.1–4.6), as were HIV positive mothers (AOR 2.1; 95% CI 1.7–2.6).

**Conclusion:**

Despite improvements to exclusive breastfeeding, failure to initiate and sustain breastfeeding is a challenge to achieving optimal breastfeeding practices. Interventions are required to address these challenges and support breastfeeding particularly among working mothers and HIV positive mothers.

## Background

Breastfeeding is the most effective preventive intervention to improve child health and development globally, and it is estimated that over 860,000 child deaths could be prevented if optimal breastfeeding practices were adopted at near universal levels [1]. Early, exclusive and sustained breastfeeding improves neonatal morbidity and mortality [2, 3]. In addition, breastfeeding protects the child against diarrhoeal disease, pneumonia and otitis media, and provides nutritional and psychosocial benefits, as well as lifelong health and socioeconomic benefits for both mother and child.

Interventions to support child nutrition have often placed the strongest focus on promoting exclusive breastfeeding (EBF) in the first 6 months of life. This is a particularly important message in high HIV prevalence countries like South Africa, where EBF for 6 months has been the recommended for HIV exposed infants since 2010, in line with World Health Organisation (WHO) recommendations [4]. When the United Nations (UN) declared 2016–2025 the UN Decade of Action on Nutrition, the breastfeeding target chosen was to improve global EBF rates to above 50% for infants aged below 6 months [5]. However, optimal breastfeeding goes beyond the first 6 months of life and includes sustained breastfeeding for 2 years and beyond, with addition of nutritious complementary feeds from 6 months of age [6]. The benefits of breastfeeding for child health, nutrition and cognitive function increase with longer durations of breastfeeding [7–9]. Thus, preventing early cessation of breastfeeding is important for child health, and for the achievement of global nutrition targets to reduce stunting and wasting [10]. However, determinants of breastfeeding practices are complex and operate at social, structural and individual levels [1].

In South Africa, previous estimates of EBF rates have been low [11, 12], and there are high rates of stunting among South African children [13]. In recent years several breastfeeding support initiatives have been implemented including mother-baby friendly initiative (MBFI) [14], kangaroo mother care (KMC), SMS support for pregnant women and mothers (MomConnect) [15], and the KwaZulu-Natal initiative for Breastfeeding Support (KIBS) described below, of which this study forms a component. Recent evidence from the KIBS baseline suggests that exclusive breastfeeding practices have improved in KZN [13], but that challenges remain for working mothers and those who are HIV positive [16].

Encouraging and supporting breastfeeding has been challenging in high HIV prevalence areas like South Africa (SA), where maternal HIV prevalence is above 40% in some areas, including KwaZulu-Natal (KZN) [17]. Frequent changes to infant feeding guidelines for HIV exposed infants over the past two decades has resulted in changing and often contradictory infant feeding messages, some of which were not supportive of breastfeeding [4, 18]. Recommendations at the time of the study were based on 2010 WHO guidelines [19], to exclusively breastfeed for 6 months and continue to breastfeed while adding complementary feeds until 12 months, as long as the mother is adherent to antiretroviral treatment (ART) and is virally suppressed [18, 20]. However, the legacy of changing messages has led to confusion among health workers and to mothers receiving confused or contradictory messages [21–23].

Returning to work is one of the biggest challenges that women face in sustaining breastfeeding. Shorter durations and lower rates of breastfeeding among working women have been shown in many settings globally, including in South Africa [16, 24–27]. A range of interventions can improve breastfeeding rates among working women, these include paid maternity leave, flexible working hours, educating mothers how to continue breastfeeding while away from their child, and facilities for lactating mothers in the workplace [28–30]. In South Africa, all women in formal work are entitled to 4 months unpaid maternity leave and those who contribute to the government unemployment insurance fund (UIF) can claim government funded maternity benefits for that period. However, these benefits exclude those who do not contribute to UIF such as teenage mothers, and women working in the informal sector [14].

We present results of two sequential surveys conducted before and after implementation of the KIBS initiative, a multipronged intervention to support breastfeeding in KZN, to assess changes in breastfeeding practices over this three-year period. We describe factors associated with EBF at 14 weeks and explore reasons for early breastfeeding cessation. In particular, we focus on changes in infant feeding practices among mothers who have returned to work or school and mothers reporting themselves HIV positive, who are at high risk of poor breastfeeding practices [16].

## Methods

### Brief description of the KIBS intervention

KIBS was a three-year project (2014–2017) which aimed to provide technical and logistical support to implement existing and proposed interventions to support breastfeeding in KZN, and was a partnership between the University of KZN and the KZN Department of Health (KZN DoH). KIBS provided support for establishment of Human Milk Banks (HMBs) in one facility in each district in KZN. In addition, project staff provided skills development, training and mentoring for health workers to support breastfeeding in clinics, hospitals, and in communities, which included skills development to support and promote breastfeeding among HIV positive mothers and among mothers returning to work or school. Posts for lactation advisors were created in all 52 hospitals providing maternity services in KZN. All lactation advisors appointed were enrolled nurses and received 2 weeks of training, followed by on-site mentoring and an assessment for competency before being deployed in the new position. A total of 58 lactation advisors were trained, of whom 51 participants completed the assessment and were deployed in 47 hospitals. Nutrition advisors are lay health workers recently recruited, trained and placed in all primary health care clinics in KZN to support nutrition. Among 571 clinic-based nutrition advisors, 555 received additional training, skills development and on-site mentoring during the KIBS project.

Finally, a media campaign was supported by KIBS to promote breastfeeding messages using radio and video messages and public service announcements.

### Study setting

KZN is the largest of 11 provinces in South Africa with a population of over 11 million people. Antenatal and postnatal services are provided at local primary health care (PHC) clinics, with almost universal coverage of antenatal care [16]. Over 90% of women deliver in a health facility, usually the district hospital, although some larger clinics perform deliveries [31]. Mothers having normal deliveries without complications are discharged within 24 h post-delivery. All maternal and newborn services are free of charge in the public healthcare system. HIV prevalence is high in KZN; over 40% of pregnant women attending government health facilities were HIV positive during the period of the study [32]. Lifelong antiretroviral treatment (ART) is available for all HIV positive women. Immunisation coverage is also fairly high in KZN, with over 80% of infants fully immunised at 1 year throughout the study period [33, 34]. Stunting rates are high in South Africa at 27% among children aged under 5 years [31], and infant mortality was estimated at 34 per 1000 live births in 2015 [35].

### Design

Two cross-sectional surveys (KIBS1 and KIBS2) were conducted at the beginning and at the end of the KIBS intervention to provide valid estimates of EBF rates in KZN at each time point, and to explore changes in infant feeding practices over the three-year period. The KIBS1 and KIBS2 surveys were conducted in PHC clinics: all fixed and mobile clinics providing immunisation services were included in the sampling frame. All mothers and child carers aged 15 years or above who attended the clinic with a baby aged 91–111 days (13- < 16 weeks) were eligible to participate. This age was chosen to coincide with the 14-week immunisation visit, with the aim of achieving a representative sample of the population. Non-maternal carers answered a subset of questions about feeding practices, but were not asked to provide detailed information about the mother’s history.

### Sample size

#### KIBS1

The sample size was calculated to provide *district level* estimates of exclusive breastfeeding rates at 14 weeks for each of the 11 districts in KZN. Nine clinics were randomly selected in each district (total 99 clinics) and 4059 interviews with caregivers were required (369 interviews per district). The sample size was calculated using the formula, n = Z2pq/d2 (where Z = 1.96 at 95% confidence; p = proportion who are exclusively breastfeeding, q = 1-p; d = absolute precision). For this study, we presumed that p < = 0.4 based on previous routine data and literature; q = 0.6; precision (d) = +/− 5%. This yielded a required sample size of 369 per district. The final sample size required was thus 369*11 = 4059 respondents. Data collection continued in all selected clinics in each district contemporaneously until the required sample was realised for that district therefore resulting in a self-weighted sample (probability proportional to size) across the clinics.

#### KIBS2

The sample size was calculated to provide only a *provincial* level estimate of exclusive breastfeeding rates at 14 weeks in KZN to identify provincial trends in feeding practices. It was not considered necessary or feasible to repeat the large-scale district level study so soon after KIBS1. A total of thirty clinics were randomly sampled, and thirty interviews were required in each clinic (a total of 900 interviews).

### Data management and analysis

Data were collected using android devices and uploaded to a centralised server in real time using proprietary software. Extensive data quality checks were conducted by a dedicated quality control team ensuring data completeness and validity.

Data cleaning and analysis were conducted using Stata 13 (StataCorp. 2013. Stata Statistical Software: Release 13. College Station, TX: StataCorp LP) to generate district level estimates of the primary variables as well as the 95% confidence intervals (95% CI). For non-normal categorical variables, we report medians and the interquartile range and where significance testing was conducted using the Wilcoxon rank sum test. For categorical variables, we report descriptive statistics and proportions using the Stata SVY adjustment to control for sampling design for KIBS1 and simple proportions for KIBS2.

In order to define categories of feeding practices, participating mothers were asked several questions about feeding practices since the baby was born. These included whether she had given any food or fluids other than breastmilk since birth, but also included specific questions about ever having given the baby formula milk or water, and about pre-lacteal feeds. Only those who consistently reported having given only breastmilk, were classified as EBF. Those who reported currently breastfeeding but who reported giving other food or fluids at any time since birth, were categorised as mixed breastfeeding. Similarly, infants attending with non-maternal caregivers were categorised as EBF if the carer reported that the infant had never been given any food or fluids other than breastmilk. Odds ratios were provided using logistic regression for comparisons of prevalence of the respective feeding modality across the two surveys. Multi-variable logistic regression was used to adjust odds ratios for demographic or social characteristics differences at the two time points. A Kaplan Meier curve and log rank test is used to analyse time to stopping breastfeeding. Hazard ratios were calculated using a Cox regression model for assessing changes in duration across the two surveys adjusted for mother returning to work or school, HIV status and urban or rural setting.

The classification of rurality utilised in this report is based on Cooperative Governance and Traditional Affairs (KZN-COGTA) definitions which segments the KZN province into 5 distinct categories [36].

## Results

A total of 4172 interviews were conducted at KIBS1 (May 2014–March 2015) and 929 at KIBS2 (Jan- August 2017), among carers bringing an infant aged 13- < 16 weeks to the clinic. Among participating carers at KIBS1, 3659 (87.8%) were mothers and 514 (12.3%) were non-maternal carers. Similarly, in the KIBS2 survey 788 (84.8%) infants attended with the mother and 141 (15.2%) with non-maternal carers. Most infants were currently living with the mother at both time points (3919/4172; 93.9% vs 875/929; 94.2%).

Characteristics of all carers, including both mothers and non-maternal carers, are shown in Table 1. Non maternal carers provided only basic information so the analysis that follows relates mainly to infants attending with the mother. At KIBS2, non-maternal carers were asked the reason for the mother’s absence, and most reported that this was because the mother was at work (64/141; 45.4%) or at school (61/141; 43.3%), otherwise the mother was sick (1.4%) or there was another reason/data was missing (9.9%). There were significant differences between participating mothers at the two time points, mothers at KIBS2 had significantly higher levels of education, were more likely to live in a household where electricity was used for cooking, were more likely to be in a relationship with the child’s father and were less likely to be receiving a child support grant for any child (Table 1). The median age of mothers was 24 years (interquartile range [IQR] 20–29) at KIBS1 and 26 years (IQR 21–31) at KIBS2, and of non-maternal carers was 32 years (IQR 24–45) and 33 years (IQR 26–44) at KIBS1 and KIBS2 respectively.

**Table 1:** Sociodemographic data for all participants

ALL CARERS	KIBS1 ***N*** = 4172 n (%)	KIBS2 ***N*** = 929 n (%)	***P*** value
**Relationship with the child**			0.12
Mother	3659 (87.7)	788 (84.8)	
Grandmother	196 (4.7)	61 (6.6)	
Father	36 (0.9)	6 (0.6)	
Other relative	203 (4.9)	59 (6.4)	
Non relative caregiver	78 (1.9)	15 (1.6)	
**Population group**			0.13
African	4094 (98.1)	898 (96.7)	
Coloured	19 (0.5)	8 (0.9)	
Indian origin	46 (1.1)	16 (1.7)	
White	13 (0.3)	1 (0.1)	
Other	0	6 (0.6)	
**Characteristics of the baby**			
Sex of baby (% male)	2045 (49.0)	472 (50.8)	0.31
**MOTHERS ONLY**			
**Mother characteristics**	***N*** **= 3659** **n (%)**	***N*** **= 788** **n (%)**	
Aged under 18 years	273 (7.5)	37 (4.7)	0.009
Has more than one child	2010 (54.9)	449 (57.0)	0.36
In a relationship with the child’s father	3244 (88.7)	740 (93.9)	**0.001**
Staying in same house as child’s father	874 (23.9)	221 (28.0)	0.10
Mother is HIV positive	1274 (34.8)	303 (38.7)	0.058
**Mothers education level**			**0.001**
None	36 (1.0)	9 (1.1)	
Completed Grade 1–7	288 (7.9)	44 (5.6)	
Completed Grades 8–11	1884 (51.5)	293 (37.2)	
Completed Grade 12 or above	1451 (39.7)	442 (56.1)	
Mother has returned to school since birth	246 (6.7)	38 (4.8)	0.061
**Income**			
Mother had returned to work since birth	296 (8.1)	65(8.2)	0.92
Receives child support grant for any child	2445 (66.8)	465 (59.0)	**0.03**
**HOUSEHOLD**			
Mother lives in rural area	2498 (68.3)	511 (64.8)	0.74
***Water source***			0.26
Piped in house/yard	2023 (55.3)	499 (63.3)	
Piped public tap	874 (23.9)	155 (19.7)	
Other safe water source (tanker truck)	63 (1.7)	26 (3.0)	
Unsafe water source	699 (19.1)	108 (13.7)	
***HH connected to electricity***			
Yes	2922 (79.9)	698 (88.6)	0.056
***Fuel used for cooking***			**0.01**
Electricity	2274 (62.1)	618 (78.4)	
Wood	1010 (27.6)	122 (15.5)	
Other (gas, paraffin, cow dung)	375 (10.3)	48 (6.1)	
***Type of toilet***			0.052
Ventilated pit latrine	1746 (47.7)	345 (43.8)	
Pit latrine	955 (26.1)	149 (18.9)	
Flush toilet in house / yard	873 (23.9)	287 (36.4)	
Bucket, Bush, No toilet	85 (2.3)	7 (0.9)	

### Feeding practices for all infants

There was some increase in the proportion of infants being exclusively breastfed among infants attending with both maternal and non-maternal carers over the three-year period between KIBS1 and KIBS2. However, this difference was larger among infants brought by the mother. The increase in EBF was largely due to a reduction in the proportion of all infants being mixed fed rather than an increase in the proportion of mothers breastfeeding. Overall, almost one-third of infants were not being breastfed at 14 weeks and this was similar at both time points. In addition, there was a large increase in non-breastfeeding among infants brought by non-maternal carers (Table 2).

**Table 2:** Feeding practices among all participating infants aged 14 weeks

	KIBS1	KIBS2	***P*** value
**ALL INFANTS**			
	***N*** **= 4172** **n (%wgt)**	***N*** **= 929** **n (%)**	
Unknown feeding practice^a^	21 (0.3)	4 (0.4)	
Not breastfeeding	1292 (31.9)	305 (32.8)	0.71
Mixed breastfeeding	881 (23.2)	151 (16.3)	**0.016**
Exclusive breast-feeding	1978 (44.6)	469 (50.5)	0.10
Overall feeding practice			**0.04**
**INFANTS ATTENDING WITH NON MATERNAL CAREGIVER**			
	***N*** **= 513** **n (%wgt)**	***N*** **= 141** **n (%)**	
Unknown feeding practice^a^	21 (2.5%)	4 (2.8)	
Not breastfeeding	338 (62.3)	113 (80.1)	**0.0020**
Mixed breastfeeding	81 (23.4)	22(15.6)	0.15
Exclusive breastfeeding	73 (11.8)	2 (1.4)	**0.0003**
**INFANTS ATTENDING WITH MOTHER**			
	***N*** **= 3659** **n (%wgt)**	***N*** **= 788** **n (%)**	
Never breastfed	346 (10.0)	57 (7.2)	0.12
Stopped breastfeeding	608 (17.0)	135 (17.1)	0.95
Mixed breastfeeding	**800 (23.1)**	**129 (16.4)**	**0.02**
Exclusive breast-feeding	**1905 (49.9)**	**467 (59.3)**	**0.02**
Overall feeding practice			**0.03**

^a^brought by non-maternal caregiver who was unable to report on current feeding practice. Significant associations shown in bold text

### Feeding practices for infants attending with the mother

#### Factors associated with EBF among participating mothers

The following analysis refers to mother-infant pairs. After controlling for the differences in participant characteristics between the two time points, the proportion of infants attending with the mother who were exclusively breastfed was higher at KIBS2 than KIBS1 (Table 3). Multi variable analysis shows that mothers who had returned to work or school and those living in a household where electricity is used for cooking were less likely to practice EBF, whereas those mothers in receipt of a child support grant were more likely to practice EBF (Table 3).

**Table 3:** Factors associated with exclusive breastfeeding multi-variable analysis among infants attending with their mothers

	EBF		Total	Unadjusted			Adjusted		
	(***n*** = 2372)		(***n*** = 4447)	OR	95CI	p	OR	95CI	p
**Study**	n	wgt%	N						
Baseline	1905	49.9%	3659	Ref			Ref		
Endpoint	467	59.3%	788	1.46	(1.1–2.0)	**0.02**	1.63	(1.2–2.3)	**0.005**
**Age group**									
< 25	1175	51.1%	2238	Ref			Ref		
25–49	1197	48.6%	2209	0.90	(0.7–1.2)	0.52	0.84	(0.6–1.2)	0.30
**Relationship with child’s father**									
No	233	41.6%	463	ref			Ref		
Yes	2139	51.1%	3984	1.47	(0.7–3.0)	0.30	1.32	(0.7–2.3)	0.34
**Living in same house as child’s father**									
No	1747	48.9%	3352	Ref			Ref		
Yes	625	52.9%	1095	1.17	(0.9–1.6)	0.28	1.20	(0.9–1.6)	0.21
**Mothers education**									
< 12	1421	50.8%	2554	Ref			Ref		
12	951	48.2%	1893	0.90	(0.7–1.2)	0.47	0.96	(0.7–1.2)	0.73
**Mother returned to school or work**									
No	2220	54.1%	3802	Ref			Ref		
Yes	152	22.2%	645	0.24	(0.2–0.4)	**< 0.001**	0.25	(0.2–0.4)	**< 0.001**
**Mother receives grant for any child**									
No	749	43.1%	1537	Ref			Ref		
Yes	1623	53.1%	2910	1.50	(1.2–1.8)	**< 0.001**	1.32	(1.1–1.6)	**0.004**
**Mothers HIV status**									
Neg	1450	50.9%	2765	Ref			Ref		
Pos	875	48.4%	1577	0.90	(0.7–1.2)	0.42	0.94	(0.7–1.2)	0.62
**Place of residence**									
Rural	1692	56.0%	3009	Ref			Ref		
Urban	680	44.0%	1438	0.62	(0.4–0.9)	**0.019**	0.73	(0.5–1.1)	0.14
**Water source**									
Unsafe	526	64.3%	896	Ref			Ref		
Piped	1846	45.7%	3551	0.47	(0.3–0.8)	**0.002**	0.65	(0.4–1.1)	0.14
**Household electricity connection**									
not connected	510	60.4%	827	Ref			Ref		
connected	1862	46.9%	3620	0.58	(0.4–0.8)	**< 0.001**	1.03	(0.6–1.7)	0.92
**Cooking fuel**									
Other	930	60.7%	1555	Ref			Ref		
Electricity	1442	42.8%	2892	0.48	(0.4–0.6)	**< 0.001**	0.60	(0.4–0.9)	**0.028**
**Type of toilet**									
Other	1821	52.8%	3287	Ref			Ref		
Flush	551	40.8%	1160	0.62	(0.5–0.8)	**0.003**	1.06	(0.7–1.6)	0.77

Significant associations shown in bold text

#### Duration of breastfeeding among mothers who stopped breastfeeding before 14 weeks

**Fig. 1:**
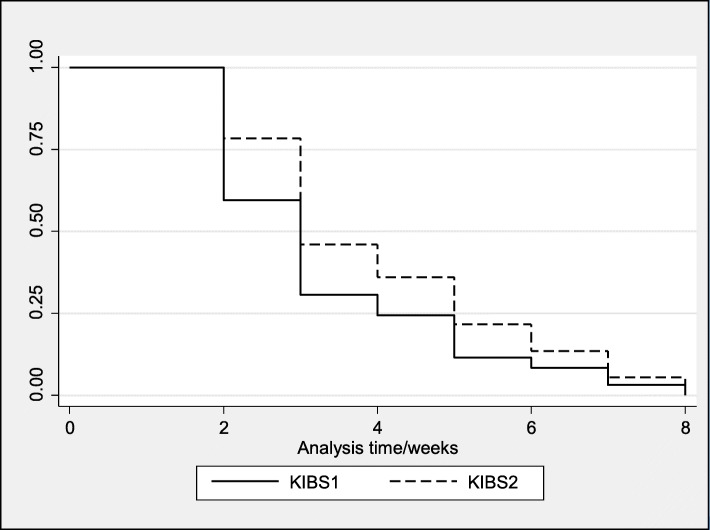
Kaplan-Meier life table curve comparing KIBS1 and KIBS2 surveys, with respect to stopping breastfeeding

At both time points a similar proportion of mothers had initiated breastfeeding but had already stopped breastfeeding at 14 weeks (17.1% vs 17.0%; *p* = 0.95). At both time points most of the mothers who stopped did so by the time the baby was 4 weeks old (69.4% KIBS1; 54.1% KIBS2), but at KIBS2 the duration of breastfeeding was longer among mothers who had stopped breastfeeding, as shown by the Kaplan Meier curve (*p* = 0.0015) (Fig. 1). Cox regression analysis further shows that the hazard ratio for stopping breastfeeding was significantly lower at KIBS2 vs KIBS1 (aHR; 95% CI 0.8 (0.6–0.9) but that no other factors were significantly associated with stopping breastfeeding (Supplementary Table 1).

#### Reasons for not breastfeeding among mothers at 14 weeks

Among infants attending with their mother a similar proportion were not breastfed at 14 weeks (either never breastfed or stopped breastfeeding) at both time points (954/3659; 26.1% vs 192/788; 24.4%; *p* = 0.30).

At KIBS2 those mothers who were no longer breastfeeding reported the primary reason for the decision to either not initiate breastfeeding and to stop breastfeeding. The commonest reason reported for *stopping* breastfeeding was that the mother had returned to work or school, and the commonest reason for *never* breastfeeding was concern about the mother’s health, including HIV. Among the 43 mothers who mentioned their own health as the main reason for stopping breastfeeding, 39 (90.7%) reported themselves HIV positive. Other common reasons for not breastfeeding were challenges related directly to breastfeeding; 54/192 (28.1%) mothers reported breastfeeding problems including problems related to the baby, to breast health and failure to establish breastfeeding (Table 4).

**Table 4:** Reasons for no longer breastfeeding at 14 weeks as reported by mothers at KIBS 2

REASON FOR NOT BREASTFEEDING	Total ***N*** = 192 n (%)	Never breastfed ***N*** = 57 n (%)	Stopped breastfeeding ***N*** = 135 n (%)
**Returning to work or school**	57 (29.7)	10 (17.5)	47 (34.8)
**Mothers health including HIV**	43 (22.4)	18 (31.6)	25 (18.5)
**Breastfeeding problems related to the baby:** includes baby hungry, not enough milk, baby crying all the time	42 (21.9)	9 (15.8)	33 (24.4)
**Breastfeeding problems related to the mother:** includes breast conditions, breastfeeding painful	7 (3.6)	2 (3.5)	5 (3.7)
**Unable to establish breastfeeding**	5 (2.6)	5 (8.8)	0 (0)
**Advised by health worker or family to stop breastfeeding**	12 (6.3)	6 (10.5)	6 (4.4)
**Other or no response**	26 (13.5)	7 (12.3)	19 (14.1)

#### Reasons for not breastfeeding: returning to work or school

At KIBS1 and KIBS2 it was common for mothers to have returned to work or school by the time the baby reached 14 weeks (542/3659; 14.8% vs 103/788; 13.1%; *p* = 0.36). At KIBS2 returning to work or school was the commonest reason reported by mothers for early cessation of breastfeeding (Table 4). Multi-variable analysis showed that after controlling for participants differences between the time points (education level; relationship with child’s father; use of electricity for cooking; receiving a child support grant) mothers who had returned to work or school were significantly more likely not to be breastfeeding at 14 weeks (AOR 3.8; 95% CI 3.1–4.6).

At KIBS1 mothers who had returned to work or school were less likely to have initiated breastfeeding than non-working or schooling mothers (19.4% vs 8.6%), but this difference was not present at KIBS2 (6.8% vs 7.3%). However, in both surveys mothers who had returned to work or school were less likely to be breastfeeding exclusively compared to non-working/schooling mothers.

Table 5 shows that the significant improvements in EBF rates achieved among non-working/schooling mothers between KIBS1 and KIBS2, are not shown among mothers who had returned to work or school. However, there are small improvements in all indicators for working/ schooling mothers. Controlling for baseline characteristics did not alter the overall findings for mothers returning to work or school.

**Table 5:** Feeding practices among mothers returning to work or school at KIBS1 and KIBS2

Feeding Practice	Mother returned to work/school since baby was born					Mother not at work or at school				
	KIBS1		KIBS2		p value	KIBS1		KIBS2		***p*** value
	(***n*** = 542)		(***n*** = 103)			(***n*** = 3117)		(***n*** = 685)		
	n	wgt%	n	%		n	wgt%	n	%	
Never breastfed	96	19.4%	7	6.8%	**0.006**	250	8.6%	50	7.3%	0.52
Stopped breastfeeding	177	34.1%	37	35.9%	0.76	431	14.4%	98	14.3%	0,98
Mixed breastfeeding	146	24.4%	30	29.1%	0.41	654	22.9%	99	14,5%	**0.01**
Exclusive breastfeeding	123	22.2%	29	28.2%	0.29	1782	54.1%	438	63.9%	**0,03**
Total	542	100%	103	100%		3117	100%	685	100%	

Significant associations shown in bold text

Further, the analysis shown in Table 5 excludes infants brought by a non-maternal caregiver, whose mothers were most often absent because of being at work or school.

#### Reasons for not breastfeeding: HIV positive

Among participating mothers, 1274/2567 (32.3%wgt) reported themselves HIV positive at KIBS1 and 303/788 (38.7%) at KIBS2. Multi variable analysis showed that after controlling for differences between the time points (education level; relationship with child’s father; use of electricity for cooking; receiving a child support grant) mothers who reported themselves HIV positive were significantly less likely to be breastfeeding at 14 weeks (AOR 2.1; 95% CI 1.7–2.6). At both time points, HIV positive mothers were significantly more likely than HIV negative mothers to report never breastfeeding and to have stopped breastfeeding at 14 weeks (Table 6).

**Table 6:** Feeding practices among HIV positive and HIV negative women at KIBS1 and KIBS2

Feeding practice	HIV positive ***n*** = 1577				***P*** value	HIV negative ***n*** = 2765				***P*** value
	KIBS1		KIBS2			KIBS1		KIBS2		
	(***n*** = 1274)		(***n*** = 303)			(***n*** = 2293)		(***n*** = 472)		
	n	%wgt	n	%		n	%wgt	n	%	
Never breastfed	212	20.1%	41	13.5%	0.15	121	5.0%	16	3.4%	0.25
Stopped breastfeeding	223	20.1%	66	21.8%	0.66	367	15.7%	66	14.0%	0.48
Mixed BF	142	11.5%	18	5.9%	**0.01**	635	28.4%	110	23.3%	0.19
Exclusive breastfeeding	697	48.4%	178	58.8%	**0.03**	1170	50.9%	280	59.3%	0.08
Total	1274	100%	303	100%		2293	100%	472	100%	

Significant associations shown in bold text

Table 6 shows that breastfeeding practices improved among HIV positive mothers between KIBS1 and KIBS2, and this improvement was in line with improvements shown among HIV negative mothers. However, the proportion of HIV positive mothers not breastfeeding at 14 weeks remained similar at both time points (435/1274; 34.1%; 40.2%wgt vs 107/303; 35.3%, *p* = 0.27). For each of the feeding practices shown in Table 6, differences remained when controlling for differences between participant characteristics between KIBS1 and KIBS2.

### Feeding advice provided to mothers

The proportion of mothers who reported receiving feeding advice in the antenatal clinic was similar at both time points (3049/3659; 83.3% vs 662/788; 84.3%, *p* = 0.74). Comparing KIBS2 with KIBS1 more mothers reported receiving *both* advice and help with breastfeeding (1809/3659; 49.4% vs 532/788; 66.4%, *p* < 0.001), and fewer mothers received neither help nor advice with feeding after delivery (624/3659; 17.1% vs 97/788; 12.3%, *p* = 0.009). Few mothers reported receiving feeding advice during a household visit from a CHW in the postnatal period at both time points (378/3659; 10.3% vs 70/788; 8.9%, *p* = 0.47).

After controlling for differences in participants characteristics, mothers who received feeding advice in the ANC were less likely to have stopped breastfeeding at 14 weeks compared to mothers who received no advice (AOR 0.71; 95% CI 0.6–0.9). Similarly mothers who received feeding advice from a CHW were less likely to have stopped breastfeeding at 14 weeks (AOR 0.75 95% CI 0.6–1.0). In contrast, mothers who received advice at the time of delivery were more likely to have stopped breastfeeding at 14 weeks compared to mothers who received no advice (AOR 1.33; 95% CI 1.1 = 1.6). However, those mothers who received both advice and help at the time of delivery were less likely to have stopped breastfeeding (AOR 0.83; 95% CI 0.7–1.0).

## Discussion

Our findings suggest that EBF rates among mothers increased significantly in KZN over the 3 years of the KIBS project, so that in 2017 almost 60% of mothers of 14 week old infants reported exclusively breastfeeding since birth, and exclusive breastfeeding among all infants was over 50%. The quality of breastfeeding improved with fewer breastfeeding mothers reporting mixed breastfeeding at 14 weeks. Further, among mothers who had already stopped breastfeeding, the duration of breastfeeding increased between KIBS1 and KIBS2. This represents a major achievement and suggests that multiple efforts to support and promote breastfeeding in KZN have been successful. Our results show the highest EBF rates reported in South Africa, but the findings are in line with improvements to EBF shown in a recent large household survey [31]. In contrast, it is important to note that the increase in exclusive breastfeeding was largely due to reduced mixed feeding among breastfeeding mothers, and no reduction was seen in the proportion of mothers not breastfeeding at 14 weeks over this three-year period. Exclusive breastfeeding was associated with not having access to electricity for cooking and not receiving a child support grant, suggesting poor socioeconomic status. Low socioeconomic status has been associated with higher breastfeeding rates in a number of African settings [37–39], and is likely to be related to the high cost of purchasing formula milk.

Achieving optimal breastfeeding goes beyond improving EBF rates, and our findings indicate that early cessation of breastfeeding is a major concern. Almost one third of infants were not breastfed at this young age, when for optimal health and development, breastfeeding should be continued for another 20 months. Sustained breastfeeding begins in the first few weeks of the baby’s life when, as our findings show, many mothers have already chosen not to breastfeed. Further, the majority of mothers still breastfeeding at 2–3 months are likely to still be breastfeeding at 1 year [31]. It is difficult, particularly in food insecure households, to provide children with adequate nutrition in the complementary feeding period if the child is no longer receiving any breastmilk [40] and stunting remains a huge problem globally, including in South Africa [31]. Achieving global nutrition targets to reduce stunting and wasting depend on sustained breastfeeding [41]. Thus, the first weeks after delivery is a critical time for interventions to support breastfeeding, so that all mothers are encouraged to initiate and continue to breastfeed for as long as possible, including those who are returning to work or are HIV positive.

Breastfeeding practices among women who had returned to work or school showed no significant improvement, contrasting with improvements shown among non-working mothers over the same time period. Further, the impact on breastfeeding of returning to work or school was substantially under-estimated since almost all non-maternal carers reported the reason for the mother’s absence was that she was at work or school, and few of those infants were breastfed. Maintaining breastfeeding while in full time work is a complex skill, which requires strong motivation from the mother, support and advice from health services and from the family and community, as well as at the work place. If there is a long distance between the mother and baby during working hours, mothers need to prepare for the return to work, and express and store breastmilk safely while working. Mothers planning to return to work or school are at high risk of early breastfeeding cessation, and identifying these mothers should be part of the routine assessment during all contacts with health services, to ensure that targeted counselling is provided. It is crucial that health workers have the skills to provide appropriate support for working mothers. Counselling messages should include the importance of initiating breastfeeding even if only for a short period, of sustaining breastfeeding even if this is not exclusive, and how to maintain breastfeeding while away from the baby. Current maternity protection does not fully comply with the requirements of the UN Convention on Elimination of Discrimination against Women (CEDAW), which has been ratified by South Africa, and requires universal access to maternity leave with pay or comparable social benefits [42]. Longer durations of statutory maternity leave, which should be paid and accessible to all women, would likely improve feeding practices. However, this must be affordable to employers and government, and must also be balanced with mothers’ socioeconomic circumstances and her right to work and provide for her family.

Our study showed significant improvements in feeding practices among HIV positive women that were in line with overall improvements in exclusive breastfeeding among all mothers, suggesting that HIV positive mothers contributed to overall improvements shown. Further, our findings suggest that changes to the infant feeding guidelines in South Africa may have led to improvement in breastfeeding rates among HIV positive women [20]. However, HIV infected mothers were still more likely to not initiate breastfeeding or to stop breastfeeding early due to concerns about their health. Studies suggest that confusion among health workers is an important legacy of the frequent changes to the infant feeding guidelines for HIV exposed infants, and that the dangers of mixed feeding are still a major concern for mothers and health workers [43]. Recent WHO guidelines (2016) adopted since this study was completed, highlight the importance of *any* breastfeeding for all mothers including those who are HIV positive, as long as they are adherent to ART [18]. This message needs to be fully adopted if HIV positive mothers are to achieve optimal feeding practices and health workers must be updated to ensure that HIV infected mother receive clear and consistent messages and support to continue breastfeeding, even if they are unable to breastfeed exclusively.

Support provided by health workers and community health workers has a huge role to play [44], and can effectively improve breastfeeding practices [1]. Our findings support other studies which show that breastfeeding challenges, including breast conditions and perceived insufficient milk, were among the important reasons for stopping breastfeeding [12, 44–46]. In our study mothers who received advice and support from health workers were more likely to be breastfeeding, and, further, the proportion of mothers receiving both advice and help during delivery increased significantly from KIBS1 to KIBS2. This may have been the result of deployment of lactation advisors in most hospitals. However, some mothers did not receive advice during antenatal care, and the proportion of mothers receiving a postnatal visit from a community health worker remained very low at both time points. Thus, opportunities for mothers to receive support for breastfeeding in the early critical weeks after the baby was born are being lost. The health system needs to address how to strengthen support for breastfeeding throughout this period, in particular routinely providing support for breastfeeding during well child or immunisation visits is essential.

### Study limitations

The major limitation of the study is that, by undertaking two cross sectional surveys with no control group, we are unable to determine the reason for the increase in exclusive breastfeeding and cannot infer that the KIBS intervention led to these improvements. However, our study does provide robust estimates of feeding practices at two time points and provides detailed information on feeding modalities with respect to characteristics such as HIV and socio-economic status. Another limitation is that the estimates are only for infants at 14 weeks, this was necessary to directly compare results at the two time points, but we are unable to assess duration of breastfeeding beyond that age. However, because of high immunisation rates at 14 weeks, and the inclusion of all carers (mothers and non-maternal carers) from the age of 15 years, our estimate may be similar to an overall population estimate. We acknowledge that some babies do not attend for immunisation at 14 weeks and were therefore excluded from our sample.

## Conclusion

Rates of exclusive breastfeeding at 14 weeks have improved significantly in KZN over a 3 year period. However, a high proportion of babies are deprived of breastmilk even at this young age, and targeted interventions aimed at improving counselling and support for all mothers, including HIV infected and working mothers, are required to address failure to initiate breastfeeding and early breastfeeding cessation.

## Supplementary information

**Additional file 1: Table S1.** Cox regression analysis showing Hazard Ratios (HR) associated with stopping breastfeeding.

## Data Availability

The data and study tools that support the findings of this study are available from the Centre for Rural Health and will be made available by the main and corresponding author on reasonable request.

## Abbreviations

ART: Ante-retroviral therapy
BREC: Biomedical Research Ethics Committee
95% CI: 95% Confidence interval
CEDAW: Convention on Elimination of Discrimination against Women
DoH: Department of Health
EBF: Exclusive breastfeeding
HIV: Human Immunodeficiency Virus
HMB: Human milk bank
IQR: Interquartile range
KIBS: KwaZulu Natal Initiative for breastfeeding support
KMC: Kangaroo mother care
KZN: KwaZulu-Natal
KZN-COGTA: KwaZulu-Natal Cooperative Governance and Traditional Affairs
MBFHI: Mother-baby friendly hospital initiative
OR: Odds Ratio
SA: South Africa
UIF: Unemployment insurance fund
UKZN: University of KwaZulu-Natal
UN: United Nations
Wgt: Weighted
WHO: World health organization
