# Supplementary material for: Are we doing enough? Improved breastfeeding practices at 14 weeks but challenges of non-initiation and early cessation of breastfeeding remain: findings of two consecutive cross-sectional surveys in KwaZulu-Natal, South Africa
Source: BMC Public Health. 2020 Apr 3;20:440. doi: 10.1186/s12889-020-08567-y (PMC7118904; doi:10.1186/s12889-020-08567-y)
Supplement: Supplementary file 1 — Additional file 1: Table S1. Cox regression analysis showing Hazard Ratios (HR) associated with stopping breastfeeding. [file 12889_2020_8567_MOESM1_ESM.docx]

**Table S1**. Cox regression analysis showing Hazard Ratios (HR) associated with stopping breastfeeding (N=699).

| **N= 699** | **n** | **HR** | **95% CI** | **p value** |
| --- | --- | --- | --- | --- |
| **Study** |  |  |  |  |
| Baseline | 590 |  |  |  |
| Endpoint | 109 | 0,79 | (0.6-0.9) | **0,03** |
| **Mother returned to work or school** | | | | |
| Not returned to work or school | 494 |  |  |  |
| Returned to work or school | 205 | 1,03 | (0.9-1.2) | 0,75 |
| **Mothers HIV status** | | | | |
| Negative | 426 |  |  |  |
| Positive | 273 | 0,97 | (0.8-1.1) | 0,71 |
| **Rural/urban residence** | | | | |
| Rural | 437 |  |  |  |
| Urban | 262 | 0,88 | (0.8-1.0) | 0,12 |
| **Water source** | | | | |
| Outside the house house | 244 |  |  |  |
| Inside the house | 455 | 0,95 | (0.8-1.1) | 0,54 |
| **Fuel used for cooking** | | | | |
| Other | 179 |  |  |  |
| Electricity | 520 | 0,94 | (0.8-1.1) | 0,46 |
| **Type of toilet** | | | | |
| Other | 479 |  |  |  |
| Flush toilet | 220 | 0,95 | (0.8-1.1) | 0,55 |

*Significant associations shown in bold text*
